# Critically ill patients with chikungunya virus infection during the carribean outbreak 2013 - 2014

**DOI:** 10.1186/2197-425X-3-S1-A348

**Published:** 2015-10-01

**Authors:** G Thiery, L Crosby, C Perreau, B Madeux, J Cossic, C Armand, C Herrmann-Storcke, F Najioullah, R Valentino

**Affiliations:** University Hospital of Guadeloupe, Intensive Care Unit, Les Abymes, France; Université des Antilles, Pointe à Pitre, France; University Hospital of Martinique, Intensive Care Unit, Fort de France, France; Department of Public Health, University Hospital of Guadeloupe, Les Abymes, France; University Hospital of Guadeloupe, Laboratory of Virology, Les Abymes, France; University Hospital of Martinique, Laboratory of Virology, Fort de France, France

## Introduction

In 2013-2014 a severe epidemic of Chikungunya fever occurred for the first time in the Caribbean and the Americas with over a million cases in 42 territories. In Guadeloupe and Martinique, the two main islands of the French West Indies, whose population is 870 000 inhabitants, an estimated 17% of the population was affected. Data on severe forms and ICU requiring cases are scarce.

## Objectives

To describe clinical, laboratory features and outcome of critically ill patients with Chikungunya.

## Methods

Patients admitted to the ICU of the university hospital of each island, with laboratory confirmed acute Chikungunya virus infection, from April to September 2014 in Guadeloupe, and from February to September 2014 in Martinique, were included.

## Results

The cohort consisted in 65 patients, their median age was 63 years (52-70), their median Simplified Acute Physiology Score II at admission was 39 (28-54). The large majority of patients had pre-existing diseases, mainly hypertension, diabetes and chronic heart failure. Twenty-seven (41.5%) patients presented with exacerbations of previous underlying disease. Neurological manifestation occurred in 17 (27.4%) patients, including six (9.2%) with Guillain-Barré syndrome, but only four (6.1%) met criteria for encephalitis. Pulmonary edema or other aggravation of chronic heart disease was present in 13 (20%) patients. Only one patient had suspected myocarditis. Twelve patients (18.7%) had severe sepsis or septic shock, including seven (10.7%) without any other clinically or microbiologically documented infection. Thirty-seven patients (56·9%) required mechanical ventilation. Hospital mortality was 27.7%.

## Conclusions

ICU requiring cases of Chikungunya fever concerned mainly patients with preexisting comorbidities, and whose medical condition brutally worsened after being affected by CHIKV. Aggravation of previous chronic heart disease and chronic renal failure are the most common clinical pictures. Apart from Guillain-Barré syndrome cases, whether severe manifestations are specifically related to CHIKV remains unclear, especially concerning central neurologic disorders and sepsis-like manifestation.

